# Photocatalytic Quantum Dot‐Armed Bacteriophage for Combating Drug‐Resistant Bacterial Infection

**DOI:** 10.1002/advs.202105668

**Published:** 2022-04-18

**Authors:** Lei Wang, Xin Fan, Mercedes Gonzalez Moreno, Tamta Tkhilaishvili, Weijie Du, Xianlong Zhang, Chuanxiong Nie, Andrej Trampuz, Rainer Haag

**Affiliations:** ^1^ Centre for Musculoskeletal Surgery Charité – Universitätsmedizin Berlin Corporate Member of Freie Universität Berlin Humboldt‐Universität zu Berlin and Berlin Institute of Health Berlin 10117 Germany; ^2^ BIH Center for Regenerative Therapies (BCRT) Berlin Institute of Health (BIH) Berlin 13353 Germany; ^3^ Department of Chemistry and Biochemistry Freie Universität Berlin Takustraße 3 Berlin 14195 Germany; ^4^ Department of Tropical Medicine and Infectious Diseases University of Rostock Rostock 18057 Germany; ^5^ Department of Orthopedics Shanghai Sixth People's Hospital Shanghai Jiao Tong University Shanghai 200233 China

**Keywords:** bacteriophage therapy, biofilm‐associated infection, functional antibacterial nanosystem, photocatalytic therapy, reactive oxygen species (ROS)

## Abstract

Multidrug‐resistant (MDR) bacterial infection is one of the greatest challenges to public health, a crisis demanding the next generation of highly effective antibacterial agents to specifically target MDR bacteria. Herein, a novel photocatalytic quantum dot (QD)‐armed bacteriophage (QD@Phage) is reported for combating green fluorescent protein‐expressing *Pseudomonas aeruginosa* (GFP‐*P. aeruginosa*) infection. The proposed QD@Phage nanosystem not only specifically binds to the host GFP‐*P. aeruginosa* while preserving the infectivity of the phage itself, but also shows a superior capacity for synergistic bacterial killing by phage and by the photocatalytic localized reactive oxygen species (ROS) generated from anchored QD components. Notably, this highly targeted QD@Phage nanosystem achieves robust in vitro antibacterial elimination for both planktonic (over 99.9%) and biofilm (over 99%) modes of growth. In a mouse wound infection model, this system also shows remarkable activity in eliminating the wound infection and promoting its recovery. These results demonstrate that the novel QD@Phage nanosystem can diversify the existing pool of antibacterial agents and inspire the development of promising therapeutic strategies against MDR bacterial infection.

## Graphical abstract

The proposed quantum dot‐armed bacteriophage (QD@Phage) can achieve robust bacterial elimination both in vitro and in vivo via phage‐assisted photocatalytic therapy (PA‐PCT). First, the nanosystem binds to the bacterial surface thanks to the preserved phage infectivity. Upon visible light irradiation, it can then locally generate singlet oxygen (^1^O_2_), thus synergistically achieving bactericidal efficacy.

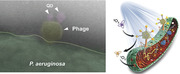

## 1 Introduction

The rapid emergence of multidrug‐resistant (MDR) bacteria, also known as “superbugs,” is endangering the efficacy of antibiotic therapy,^[^ ^1^ ^]^ and it is predicted that MDR bacterial infections will kill over 10 million people annually by 2050 due to the shortage of active antibiotics.^[^ ^2^ ^]^ MDR *Pseudomonas aeruginosa* (*P. aeruginosa*) infections are of particular concern because they often result in chronic wounds and are the leading causes of hospitalizations, disabilities, and deaths worldwide. This growing health crisis is mainly caused by the frequent misuse and overuse of antibiotics, especially broad‐spectrum antibiotics, which lack specific bacterial targeting ability. On top of that, the formation of biofilm, which is complex clusters of bacteria, merged by extracellular polymeric substances (EPS), results in a further increase in bacterial tolerance to antibiotics by three orders of magnitude.^[^ ^3^ ^]^ In contrast, the development of new drugs is rather slow, with only a few new antibiotics approved to treat “superbugs” in the past several decades.^[^ ^4^ ^]^ Therefore, it is of critical necessity to develop alternative antibacterial therapeutic approaches to control MDR bacterial infections.

As a natural predator of bacteria, bacteriophages (phages), offer hope as a promising alternative treatment for MDR bacteria since they show a different bactericidal mechanism than antibiotics.^[^ ^5^ ^]^ Upon infecting host bacteria, the phages hijack the bacterial machinery to produce their progenies,^[^ ^6^ ^]^ which eventually induce bacterial lysis and infect adjacent bacteria. In the biofilm, some phages encoding EPS‐degrading enzymes might be particularly useful against biofilms. A diverse group of phage‐encoded enzymes, called depolymerases, capable of degrading EPS involved in the biofilm matrix in order to promote phage diffusion through the biofilm has been described,^[^ ^7^ ^]^ ultimately causing great damage to the biofilm. Furthermore, thanks to their high host specificity, phages do not affect the skin microflora.^[^ ^8^ ^]^ However, due to their high mutative tendency, host bacteria can develop resistance against phages, subverting their bactericidal action.^[^ ^9^ ^]^ Therefore, it is difficult for mono‐phage therapy to achieve bacterial eradication.^[^ ^10^ ^]^ Different strategies have been studied to improve successful treatments, especially against biofilm infections, such as combination with antibiotics.^[^ ^7^ ^]^ Nowadays, new concepts are emerging in the design of phage‐based treatments to maximize the therapeutic efficacy of phage, where phage functionalization might have a great potential.

Nanomaterials have been developed as alternatives to antibiotics in fighting bacterial infections. A range of nanomaterials has attracted interest as photosensitizers that can offer antimicrobial behavior for photocatalytic therapy (PCT).^[^ ^11^ ^]^ Especially, quantum dots (QDs) have received extensive attention as a photosensitizer for PCT,^[^ ^12^ ^]^ owing to their advantages of ultrasmall size (5–25 nm) for loading into multifunctional systems, high photocatalytic efficiency for bacterial elimination,^[^ ^13^ ^]^ and inherent fluorescence emission for acting as imaging probes to detect bacteria.^[^ ^14^ ^]^ Upon light irradiation, these QDs can generate reactive oxygen species (ROS) that contribute to highly oxidative damage of cellular substances such as cell membranes and nucleic acids.^[^ ^15^ ^]^ Yet, the ultrashort lifetime and diffusion distance of ROS remain significant challenges, resulting in greatly compromised antibacterial efficacy due to quantum dots' inability to recognize bacteria. Meanwhile, nonspecific ROS production over the course of treatment can cause great damage to healthy tissues near the infection site.^[^ ^16^ ^]^ Clearly, targeting photosensitizer to the MDR bacteria is crucial to overcoming the shortcomings of today's QD‐based PCT and promoting its further application. To the best of our knowledge, no studies to date have reported a strategy of synergistic phage‐assisted PCT (PA‐PCT).

**Figure 1:**
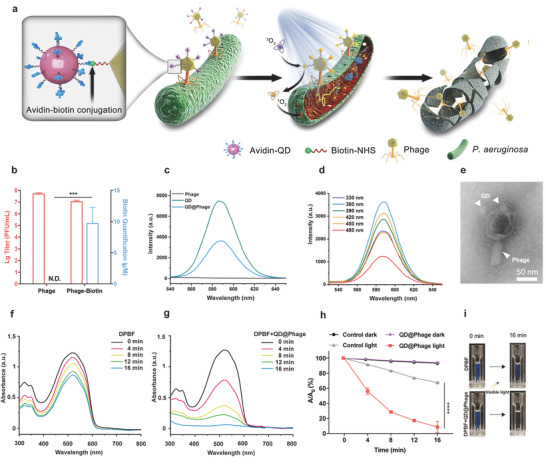
Synthesis and photocatalytic performance of QD@Phage. a) Schematic illustration of phage‐assisted photocatalytic therapy against GFP‐*P. aeruginosa*. b) Phage titer and biotin quantification after optimal dropping dose of Biotin‐NHS. N.D. represents no detection. The data are presented as mean ± standard deviation (SD), *n* = 3. c) Fluorescence spectra of phage, QD, and QD@Phage at excitation wavelength 360 nm. d) Fluorescence spectra of QD@Phage with the excitation wavelength from 330 to 480 nm. e) A representative transmission electron microscope (TEM) image of the QD@Phage. Scale bar: 50 nm. UV–vis absorption spectra of f) 1,3‐diphenylisobenzofuran (DPBF) and g) DPBF + QD@Phage with visible light irradiation, respectively. h) Time‐dependent bleaching of DPBF by QD@Phage with or without exposure to visible light. *A*_0_ is the initial absorbance of DPBF, and *A* indicates the residual absorbance in solution at time t. The PBS treatment served as control group. i) Photographs of DPBF, in the absence and presence of QD@Phage, under 16 min of visible light irradiation. The data are presented as mean ± SD, *n* = 3. ***p < 0.001 and ****p < 0.0001 by *t*‐test for data in (b) and (h).

Herein, we report a novel QD@Phage hybrid nanosystem that combines the advantages of phage‐based therapies and PCT. Using avidin‐biotin bioconjugation, a Cd‐based QD is successfully conjugated to a phage that targets green fluorescent protein‐expressing *P.aeruginosa* (GFP*‐P. aeruginosa*).^[^ ^17^ ^]^ By virtue of the phage component, the phage first assists the QD in locating on the surface of bacteria, and then, upon light irradiation, the generation of singlet oxygen (^1^O_2_), a kind of ROS, destroys the host GFP‐*P. aeruginosa* via PA‐PCT (**Figure** **1**a). In a mouse wound model, as a result of PA‐PCT, the QD@Phage functioned as a “precision‐guided killer”, which significantly reduced the bacterial colonization and accelerated the wound healing process without leading to inflammation. We anticipate the novel nanosystem with highly specific targeting, rapid photocatalytic antibacterial efficacy, and good biosafety has a great promise as a candidate for next‐generation antimicrobial therapies.

## 2 Results and Discussion

### 2.1 Synthesis and Characterization of QD@Phage Nanosystem

The QD@Phage nanosystem was prepared by avidin‐biotin conjugation (Figure 1a). Briefly, the phage, whose genome is shown in Figure S1 (Supporting Information), was first biotinylated (Phage‐biotin) using *N*‐hydroxysuccinimidobiotin (Biotin‐NHS), then streptavidin‐coated Cd‐based QD (QD) was anchored onto the biotinylated phages via bioconjugation reaction. Figure 1b shows that the phages maintained high infectivity with a negligible titer decrease after biotinylation. However, when the Biotin‐NHS linker amount was beyond 9.78 µM, a sharp decrease in phage titer was witnessed, because the overdoses linkers may also anchor the phage tail fibers, which play crucial roles in host bacterial recognition, and eventually impair phage infectivity. Therefore, 9.78  µM was chosen as the optimized biotinylation condition (Figure S2, Supporting Information). Next, the fluorescence emission profile of the QD@Phage (Figure 1c,d) was investigated to verify whether the QD had been successfully anchored onto the phages. The QD@Phage exhibited a fluorescence emission peak at 590 nm when excited at 360 nm; the spectra were identical to pristine QD, implying that the QD had been successfully anchored upon the surface of the phages. Moreover, QD@Phage displayed a wide excitation range, but with a stable emission wavelength at 590 nm, demonstrating that it is an excellent imaging agent. Collectively, the fluorescence emission properties of QD@Phage convince us of its potential for targeting and imaging bacteria. By transmission electron microscope (TEM), the unique structure of QD@Phage was also observed (Figure 1e). Meantime, the pristine phage without functionalization is shown in Figure S3, Supporting Information. Besides, the QD@Phage nanosystem presented in our study is in principle phage‐independent and could be adapted to other types of phages, as the vast majority of phages exhibit an outermost protein layer composed of proteins which are in turn composed of long chains of amino acid subunits, which display primary amine (−NH_2_) groups on their N‐terminus, necessary to conjugate the Biotin‐NHS linker.

Then, to validate the photocatalytic performance of QD@Phage, 1,3‐diphenylisobenzofuran (DPBF) was used as a probe to detect the ^1^O_2_ generation of QD@Phage.^[^ ^18^ ^]^ Both groups showed typical absorbance of DPBF at 510 nm at 0 min (Figures 1f,g, and S4, Supporting Information), presenting apparent blue fluorescence in the cuvettes (Figure 1i). The absorbance of the DBPF solution with QD@Phage at 510 nm decreased by 85% after 16 min light irradiation (Figure 1h). The solution also turned from blue to colorless during this process, indicating the depletion of ^1^O_2_ by DPBF. Without light excitation, both QD@Phage and the control group exhibited a negligible change in absorbance over a 16‐min incubation (Figure S4, Supporting Information), suggesting a lack of ^1^O_2_ generation. These results reveal that the QD@Phage possesses robust photocatalytic ^1^O_2_ production ability. We assume the combined capability of the QD@Phage, including inherent bacterial recognition by phage and robust photocatalytic performance by QD, highlights the potential of this nanosystem as an antibacterial agent via PA‐PCT.

### 2.2 In Vitro Antibacterial Activity of QD@Phage Nanosystem

**Figure 2:**
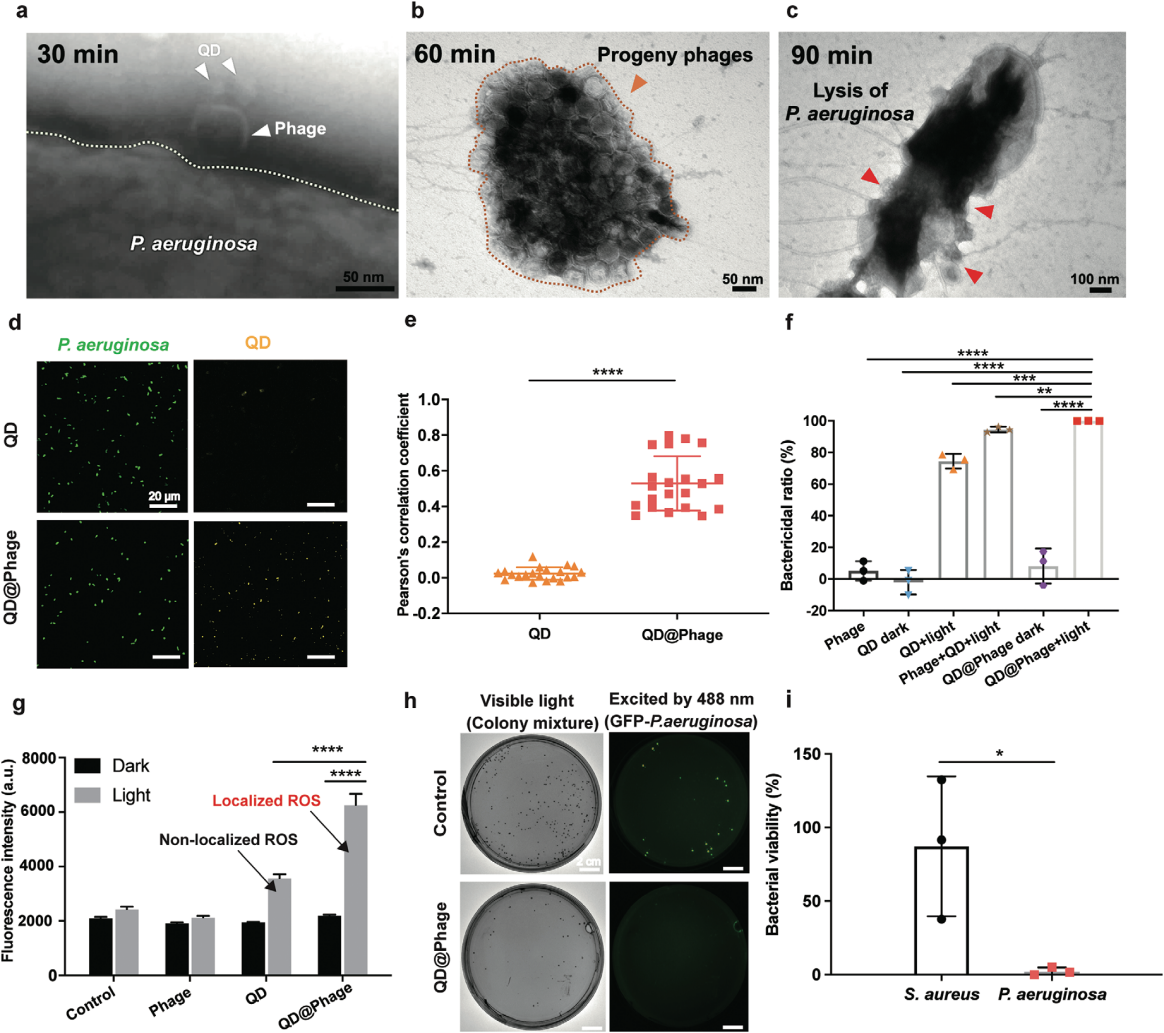
In vitro antibacterial profile of QD@Phage. TEM images revealing PA‐PCT processes of QD@Phage: a) host bacteria binding, b) progeny phages releasing, and c) lysis of a bacterium. Scale bar: 50 nm in (a) and (b); 100 nm in (c). d) Confocal scanning microscopy (CLSM) images of QD and QD@Phage treated GFP‐*P. aeruginosa*. GFP‐*P. aeruginosa* (green fluorescence); QD (yellow fluorescence). Scale bar: 20 µm. e) Colocalization analysis as determined by the Pearson's correlation coefficient statistically analyzed from CLSM images. The data are presented as mean ± SD, *n* = 20. f) Bactericidal ratios for phage, QD, phage+QD, and QD@Phage against GFP‐*P. aeruginosa* under different conditions after 90 min of incubation. The data are presented as mean ± SD, *n* = 3. g) Intracellular ROS level of GFP‐*P. aeruginosa* after co‐incubation with phage, QD, and QD@Phage, with or without light irradiation, for 90 min using 2′,7′‐dichlorodihydrofluorescein diacetate (DCF‐DA) assay kit. The data are presented as mean ± SD, *n* = 3. h) Specific antibacterial test of QD@Phage by imaging of GFP‐*P. aeruginosa* and MRSA, with visible light representing a mixture of bacteria, and the green fluorescence excited by 488 nm irradiation representing the GFP‐*P. aeruginosa*. Scale bar: 2 cm. i) Bacterial viability of GFP‐*P. aeruginosa* and MRSA after the QD@Phage light treatment. The PBS treatment served as the control group in all experiments. The data are presented as mean ± SD, n = 3. **p < 0.01; ***p < 0.001; ****p < 0.0001 by one‐way ANOVA followed by Dunnett's post hoc test for data in (e–g), *p < 0.05 by *t*‐test for data in (i).

For the antibacterial activity study, we first used TEM to monitor the PA‐PCT process. As shown by the phage adsorption rate in Figure S5 (Supporting Information), after 30 min of incubation, the majority of QD@Phage had targeted and aggregated to the host cells. Therefore, we decided to apply light irradiation after 30 min of incubation in the following tests. As evidenced also by TEM (**Figure** **2**a), after a 30 min incubation, QD@Phage was successfully located on the surface of the GFP‐*P. aeruginosa*. Once light irradiation was applied, after another 30 min of incubation, we observed a cluster of progeny phages that were going to release from the lysed bacteria (Figure 2b). Finally, as a result of PA‐PCT, we found a large number of lysed bacteria after incubating for 90 min (Figure 2c), which could be attributed to the synergistic bactericidal effect of phage and QD‐generated ^1^O_2_ against GFP‐*P. aeruginosa*.

We then used confocal scanning microscopy (CLSM) to test the host bacterial recognition and imaging ability of QD@Phage. As shown in Figure 2d, the GFP‐*P. aeruginosa*, with inherent green fluorescence, was labeled in yellow by QD@Phage after a 30 min incubation, whereas only a weak yellow signal was detected in the QD‐treated group. By investigating Pearson's correlation coefficient between the green and yellow channels, we found that the QD@Phage‐treated group showed a remarkably higher colocalization correlation as compared to the QD‐treated group. This finding demonstrates that QD@Phage possesses robust host bacterial recognition and imaging abilities (Figure 2e).^[^ ^19^ ^]^

We further investigated the bactericidal activity of QD@Phage against GFP‐*P. aeruginosa* by plate‐counting (Figure 2f and Figure S6, Supporting Information). In this experiment, phage, QD, mixture of phage, and QD treated groups were compared with QD@Phage. QD@Phage and QD with visible light irradiation, each exerted a significantly higher bactericidal effect as compared to their application under the dark condition and to the action of the phage alone; this is due to the photocatalytic ^1^O_2_ production ability of QD.^[^ ^20^ ^]^ Notably, by imposing PA‐PCT on bacteria, QD@Phage displays robust bacteria‐killing properties (over 99.9%) after a 30 min incubation and a 60 min visible light irradiation. Moreover, we evaluated the antibacterial efficiency of QD@Phage by a time‐killing assay, as shown in Figure S7 (Supporting Information). Meanwhile, a mixture of phage and QD under light irradiation demonstrated lower antibacterial activity (94.54%) than QD@Phage under light irradiation, which suggests that localizing ROS on the surface of bacteria played a critical role in achieving high bactericidal efficacy. Overall, both the QD dark group and the phage group showed relatively weak destructive effects.^[^ ^21^ ^]^ Also, in comparing QD@Phage with gentamicin, we found that QD@Phage showed a comparable bactericidal effect and a significantly higher bactericidal efficiency at an earlier incubation time (Figure S8, Supporting Information).

We also semiquantitatively measured the bacterial intracellular ROS level when treated with phage, QD, and QD@Phage by using a 2′,7′‐dichlorodihydrofluorescein diacetate (DCF‐DA) assay kit. The analysis revealed a significantly higher ROS level within the bacterial cells after treatment by QD@Phage light (Figure 2g) as compared to the QD light group, suggesting that the localized ROS generated by QD@Phage caused greater oxidative stress than the non‐localized ROS generated by the QD light group.

To investigate whether the nanosystem can selectively target the host bacteria, we applied QD@Phage in a polymicrobial condition with GFP‐*P. aeruginosa* (G−) and MRSA (G+) to investigate its bacterial killing activity by observing the colonies in bright‐field and fluorescent images after 90 min coincubation (Figures 2h,i). Nearly all of the GFP‐*P. aeruginosa* were successfully killed, with no colony observed when excited by 488 nm irradiation, whereas a great number of MRSA colonies were still witnessed, indicating the high selective capacity of the QD@Phage system towards the targeted bacteria (GFP‐*P. aeruginosa*). Besides, QD@Phage showed infectivity stability comparable to phage (Figure S9, Supporting Information), which guarantees its long‐term application.

### 2.3 In Vitro Antibiofilm Activity of QD@Phage Nanosystem

**Figure 3:**
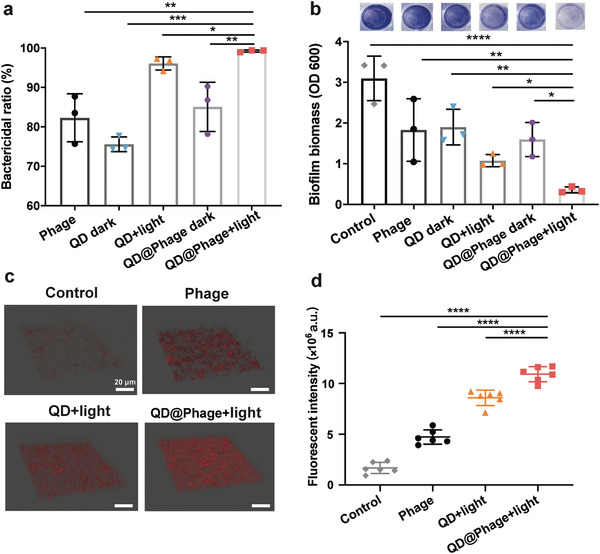
In vitro antibiofilm activity of QD@Phage. a) The biofilm bactericidal ratio of phage, QD, and QD@Phage under different conditions after 24 h of incubation. The data are presented as mean ± SD, n = 3. b) Biofilm biomass quantification using crystal violet staining after various treatments. The data arepresented as mean ± SD, *n* = 3. c) Confocal microscopy images of dead cells in the biofilm structure after treatments by phage, QD, and QD@Phage under different conditions. Dead cells are labeled in red with propidium iodide (PI). Scale bar: 20 µm. d) Semiquantitative statistics of dead cells' fluorescence intensity in the biofilm structure in (c). The PBS treatment served as a control group in all experiments. The data are presented as mean ± SD, *n* = 6. *p < 0.05; **p < 0.01; ***p < 0.001; ****p < 0.0001 by one‐way ANOVA followed by Dunnett's post hoc test for data in (a), (b), and (d).

The formation of biofilm is the major cause of bacterial chronic disease, since it endows bacteria with additional resistance to antimicrobials.^[^ ^22^ ^]^ We therefore evaluated the antibiofilm efficacy of QD@Phage against mature GFP‐*P. aeruginosa* biofilm by determination of GFP‐*P. aeruginosa* biofilm colony counts and biofilm biomass. As shown in **Figure** **3**a and Figure S10 (Supporting Information), the QD@Phage light group showed significantly enhanced antibiofilm properties as compared to other groups, with a bactericidal rate of 99.24%. Meantime, we evaluated the anti‐biofilm efficiency of QD@Phage by the time‐killing assay, as shown in Figure S11 (Supporting Information). Additionally, under crystal violet staining, this group showed the lowest amount of biofilm biomass in the well (Figure 3b, top‐view), demonstrating that the PA‐PCT using QD@Phage caused great damage to the biofilm structure, whereas all other groups showed relatively low antibiofilm activity. Moreover, the biofilm bactericidal ratio of phage, QD light, and QD@Phage light under different dosages was also measured (Figure S12, Supporting Information), suggesting a dose‐dependent efficacy in the tested groups.

Meanwhile, we used CLSM to observe the 3D biofilm structure and dead bacteria in different treatment groups. The alive bacteria in all groups showed a similar intensity of GFP fluorescence (Figure S13, Supporting Information), whereas the dead cells specifically labeled in red by propidium iodide (PI) displayed a significantly different intensity (Figure 3c). Upon treatment with QD@Phage light, representative images and statistical analysis (Figure 3d) revealed that the majority of bacterial cells within the biofilm exhibited apparent red fluorescence compared to other tested groups, implying that QD@Phage can also significantly and effectively kill sessile cells. Taken together, these findings indicate that QD@Phage shows robust antibacterial and antibiofilm activity in vitro; we therefore envision that it may also serve in vivo as an antibacterial agent for the treatment of bacterial infections.

### 2.4 In Vivo Assessment of Wound Sterilization and Healing

**Figure 4:**
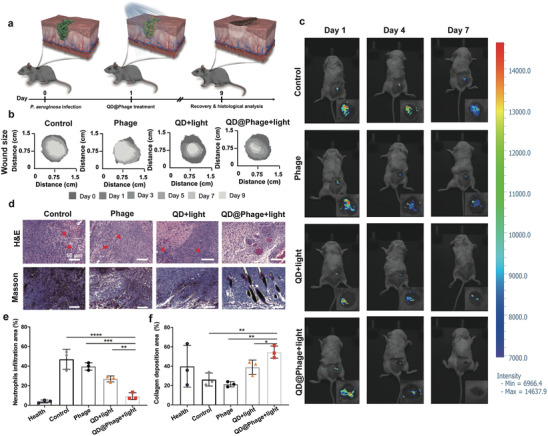
In vivo antibacterial profile of QD@Phage. a) Schematic illustration of mouse wound infection model and the process of treatment with QD@Phage. b) Traces of wound area after 9 days of various treatments. c) Representative fluorescence imaging signals for GFP‐*P. aeruginosa* in the wound during treatment. d) Representative histological photomicrographs of epidermal sections of GFP*‐P. aeruginosa*‐infected wound after various treatments, with Hematoxylin and Eosin (H&E) and Masson staining. Red arrows highlight typical neutrophils. Scale bar: 50 µm. Percentage of e) neutrophil infiltration area and f) collagen deposition area after various treatments, based on corresponding images of H&E staining and Masson trichrome staining. The PBS treatment served as control group. The data are presented as mean ± SD, *n* = 3. *p < 0.05; **p < 0.01; ***p < 0.001; ****p < 0.0001 by one‐way ANOVA followed by Dunnett's post hoc test for data in (e) and (f).

Before carrying out the in vivo antibacterial study, cytotoxicity assays showed that QD@Phage had good cytocompatibility in human A549 and human keratinocyte (HaCat) cell lines (Figure S14, Supporting Information). We then developed a mouse wound infection model using GFP*‐P. aeruginosa* to evaluate the efficiency of QD@Phage light on in vivo anti‐infective therapy. The QD@Phage with light treatment procedure is illustrated in **Figure** **4**a. In brief, a small round incision (1cm in diameter) on the dorsal epidermis of mice was first infected for 24 h with 100 µL of GFP*‐P. aeruginosa* (1 × 10^8^ CFU mL^−1^) to establish the in vivo wound infection model. Afterwards, 100 µL of QD@Phage (10^6^ PFU mL^−1^, only one dosage over the entire treatment) was directly applied to the infected wounds. 30 min later, visible light irradiation was introduced to begin PA‐PCT for wound disinfection. Eventually, granulation tissue was regenerated and deposited from the dorsal dermis tissue, and the wound tissue formed scabs and exfoliated.

We used digital photography to record the wound healing process at different timepoints (Figure S15, Supporting Information). On day 1, we observed that the infected wounds were all surrounded by inflamed epidermis with sanies, demonstrating successful infection with GFP*‐P. aeruginosa*. Representative traces of wound areas over 9 days are shown in Figure 4b; the corresponding therapeutic effect was analyzed using the wound closure rate (Figure S16, Supporting Information). On Day 9, QD@Phage with visible light irradiation showed a closure ratio of 91.2% of the original wound area, demonstrating that treatment by QD@Phage with visible light irradiation can effectively eliminate GFP*‐P. aeruginosa* infection and accelerate wound healing. The phage and QD light groups with suppuration presented much lower closure ratios than QD@Phage light (59.8% and 76.3%, respectively), suggesting delayed wound healing.

In vivo fluorescence imaging of the infected wounds revealed bacterial intensity during treatment (Figure 4c).^[^ ^23^ ^]^ We noticed very large areas of high fluorescence intensity on day 1, which again indicated the successful in vivo establishment of GFP*‐P. aeruginosa* wound infection. For the QD@Phage light‐treated group, both the area and intensity of fluorescence decreased dramatically over time, and by day 7 no intensity was detected, suggesting that the bacteria had been successfully killed in the wound sites.^[^ ^24^ ^]^ On the contrary, the other groups still showed fluorescence after 7 days of treatment, indicating the continued existence of GFP‐*P. aeruginosa* infection, fostering a prolonged inflammation state of the wound, which could be the reason for a slower healing process.

To explore the inflammation and healing status of the wound, we further investigated the wound areas using H&E and Masson staining (Figure 4d); the corresponding semiquantitative analyses are shown in Figures 4e,f. No significant infiltration of neutrophils was observed after QD@Phage light treatment, which was comparable to healthy skin tissue (Figure S17, Supporting Information). Apparent lobulated neutrophil infiltration (indicated by red arrows in Figure 4d) was seen in other groups, demonstrating an apparent sign of continued bacterial infection. The collagen fibrils were analyzed by Masson staining (Figure 4f), and the wounds of the QD@Phage light group exhibited well‐established collagen fibers and dermal layers after 9 days of treatment. Furthermore, an abundance of dead cellular debris and areolar connective tissues with disordered texture were noticed in the rest groups, indicating a delayed tissue regeneration process. The above results add excellent antibacterial properties to PA‐PCT's roster of advantages, confirming that one dose of QD@Phage with visible light irradiation offers great promise for treating MDR bacterial skin infections. For one thing, a single dose of the QD@Phage demonstrates significant bacterial reduction when exposed to visible light as we discussed before, for another thing, the progeny phage from QD@Phage can keep killing the bacteria in the remaining days to promote wound healing.

## 3 Conclusion

In summary, we have successfully synthesized a QD@Phage nanosystem for combating bacterial infections. In a unique type of phage‐assisted photocatalytic therapy, termed PA‐PCT, QD@Phage can target host bacteria via the inherent infectivity of phages, and upon visible light irradiation, the QD can locally generate massive ROS to further enhance bactericidal activity. In vitro experiments showed that QD@Phage efficiently eliminated planktonic bacteria (by over 99.9%) with good cytocompatibility. More surprisingly, it showed highly efficient antibiofilm activity (over 99%) and can achieve safe and robust skin wound healing. The data above demonstrate the proposed QD@Phage nanosystem's promising therapeutic effects: robust bacterial disinfection capability for targeting and eradicating MDR bacterial infections. Our findings also provide a new perspective for the development of novel catalytic antibacterial nanoplatforms with high specificity. Notably, limited from the short tissue penetration depth of visible light, the QD@Phage is still facing challenges in treating deep bacterial infections, but it is expected that by using our proposed synthetic method, phages conjugated with near‐infrared laser‐ or sono‐sensitizers can also be successfully fabricated and applied for treating deep tissue bacterial related infections in the near future.

## 4 Experimental Section

### Synthesis of QD@Phage

The synthesis of QD@Phage includes two main steps: phage biotinylation and bioconjugation between biotinylated phage (Phage‐biotin) and streptavidin‐coated quantum dot (QD). For phage biotinylation, typically, 300 µg of Biotin‐NHS was dissolved in 40 µL of Dimethyl sulfoxide (DMSO) to prepare a biotinylation solution, then the biotinylation solution was introduced to 1 mL of purified phages solution (10^7^ PFU mL^−1^ in PBS, pH≈8), and the whole biotinylation process lasted overnight under room temperature with mild stirring. After removing the excess Biotin‐NHS by dialysis for 24 h two times at 4 °C, Phage‐biotin was collected for subsequent use. For bioconjugation, typically, 10 µL of QD aqueous (1 µM) was mixed with Phage‐biotin for overnight under room temperature with mild stirring, the residual QD was removed by centrifugation using an Ultrafree‐MC Centrifugal Filter (Merck), finally, the QD@Phage was collected for further use.

## Conflict of Interest

The authors declare no conflict of interest.

## Author Contributions

L.W. and X.F. contributed equally to this work. C. N., X. F., L. W., A. T., and R. H. conceived the idea for this project. X. F., L. W., and C. N. assisted with the figure layout and scheme design. X. F., L. W., C. N., M. G. M., T. T., and X. L. Z discussed the manuscript structure and modified figures. L. W. and X. F. synthesized the QD@Phage. L. W., M. G. M, and T. T. performed bacterial/biofilm binding study and in vitro and in vivo antibacterial/antibiofilm tests. X. F. measured the catalytic performance of the QD@Phage and performed CLSM observation. W. J. D. performed cytocompatibility experiment and analyzed the data. The manuscript was written through the contributions of all the authors. All authors have approved the final version of the manuscript.

## Supporting information

Supporting Information

## Data Availability

The data that support the findings of this study are available from the corresponding author upon reasonable request.
