# Supplementary material for: Photocatalytic Quantum Dot‐Armed Bacteriophage for Combating Drug‐Resistant Bacterial Infection
Source: Adv Sci (Weinh). 2022 Apr 18;9(17):2105668. doi: 10.1002/advs.202105668 (PMC9189633; doi:10.1002/advs.202105668)
Supplement: Supplementary file 1 — Supporting Information [file ADVS-9-2105668-s001.pdf]

ADVANCED SCIENCE
Open Access

Supporting Information

for *Adv. Sci.*, DOI 10.1002/advs.202105668

Photocatalytic Quantum Dot-Armed Bacteriophage for Combating Drug-Resistant Bacterial Infection

Lei Wang, Xin Fan, Mercedes Gonzalez Moreno, Tamta Tkhilaishvili, Weijie Du, Xianlong Zhang, Chuanxiong Nie, Andrej Trampuz* and Rainer Haag**

Supporting Information

©Wiley-VCH 2022

69451 Weinheim, Germany

Photocatalytic Quantum Dot-Armed Bacteriophage for Combating Drug-Resistant Bacterial Infection

Lei Wang [1] [3]†, Xin Fan [2] [3]†*, Mercedes Gonzalez Moreno [1] [3], Tamta Tkhilaishvili [1] [4], Weijie Du [3], Xianlong Zhang [5], Chuanxiong Nie [2], Andrej Trampuz [1] [3]*, Rainer Haag [2]*

[1] Centre for Musculoskeletal Surgery, Charité – Universitätsmedizin Berlin, Corporate Member of Freie Universität Berlin, Humboldt-Universität zu Berlin, and Berlin Institute of Health, Berlin, Germany

[2] Department of Chemistry and Biochemistry, Freie Universität Berlin, Takustraße 3, 14195 Berlin, Germany

[3] BIH Center for Regenerative Therapies (BCRT), Berlin Institute of Health (BIH), Berlin, Germany.

[4] Department of Tropical Medicine and Infectious Diseases, University of Rostock, Rostock, Germany

[5] Department of Orthopedics, Shanghai Sixth People's Hospital, Shanghai Jiao Tong University, 200233 Shanghai, China

†These authors contributed equally to this work.
* Corresponding authors: xinf94@zedat.fu-berlin.de; andrej.trampuz@charite.de; haag@zedat.fu-berlin.de

Supporting Information

Materials

The GFP-expressing *Pseudomonas aeruginosa* (ATCC® 10145™), MRSA (ATCC® 43300™), human A549 and human keratinocyte (HaCat) cell lines were obtained from the American Type Culture Collection (ATCC); A novel lytic bacteriophage infecting GFP-*P. aeruginosa* was isolated from hospital sewage. Cd-based Core/Shell Quantum Dots with Streptavidin (OCNQSS525) and Biotin-NHS (203112), dimethyl sulfoxide (DMSO, 99.7%), Crystal violet (1092180500), 1,3-diphenylisobenzofuran (DPBF, 105481) and Masson kit (HT1) were purchased from Sigma-Aldrich; Ultrafree-MC Centrifugal Filter was purchased from Merck; Cell Counting Kit-8 (ab228554) and H&E Staining Kit (ab245880) were purchased from Abcam. EZ-Link® Biotin-NHS Reagents (20217), Pierce™ Biotin Quantitation Kit (28005) propidiumiodid (P1304MP) and 2′,7′-dichlorodihydrofluorescein diacetate assay kit (DCF-DA) were purchased from Thermo Scientific.

Material characterizations

The morphological characterization of QD@Phage and phage was performed using a transmission electron microscope (TEM, Leo TEM 906, ZEISS). Ultraviolet-Visible spectra was measured on UV/vis Spectrometer Agilent Cary 8454 and fluorescent spectra was measured on plate reader (Tecan, Infinite M200 Pro). A 1-cm path length quartz cuvette was used for singlet oxygen detection at room temperature. Fluorescence images *in-vivo* were measured by a VISQUE InVivo Smart-LF (Vieworks, Anyang, South Korea).

Annotation and bioinformatic analysis of phage genomes

Sequencing was performed on an Illumina MiSeq instrument equipped with a nanoFlowcell (Illumina MiSeq Reagent Nano Kit v2, Brussels, Belgium, paired-end 2*250 bp reads). The phage genome was assembled using the SPAdes-based PATRIC genome assembly v3.6.1261. The closest similar phage was retrieved using BLASTn v2.13.063. Genome alignment to these identified phages was performed using MEGA11. Resulting aligned phage genomes were functionally annotated through the RASTtk pipeline and manually curated using the BLASTp program v2.13.0. Genbank files were finalized using Artemis v18.1.0. To visualise and illustrate phage genome, a circular representation was created using

Proksee (https://proksee.ca/). The databases of CARD, Resfinder, PlasmidFinder, and VFDB were used to exclude the undesirable genes.

Biotinylation of phage

Typically, phage solution (10^7 PFU/mL, 1 mL in pH~8 PBS) was incubated with Biotin-NHS (feeding dose: 878.79 μM, 40 μL in DMSO) overnight. Afterwards, the unreacted Biotin-NHS was removed via dialysis and the Phage-biotin was ready for further use. Also, Phage-biotin 1 and phage-biotin 2 were synthesized with different feeding doses of 87.87 μM and 1757.6 μM of Biotin-NHS, respectively, which were used as comparisons. The biotin amount of Phage-biotin (10^6 PFU/mL, 1 mL) was further quantified by Pierce™ Biotin Quantitation Kit according to manufacturer's instructions. Meanwhile, the double layer agar plaque assay is employed for enumeration of phage titer.

Transmission electron microscopy

For transmission electron microscopy observation, 10 μL of QD@Phage solution (10^6 PFU/mL) was loaded on a copper grid with carbon support layer 45 s, and the excess solution was removed using a filter paper. Afterwards, the grid was negatively stained with 2% uranyl acetate for 45 s and washed three times with distilled water. Finally, the grid was air-dried and ready for morphology observation. Moreover, to monitor the bacterial killing process upon PA-PCT treatment, the solution of QD@Phage incubated with host bacteria under different protocols (30 min with dark; 30 min with dark + 30 min with light; 30 min with dark + 60 min with light) were dripped on copper grids. Afterwards, all grids were stained, washed and observed with the same protocol.

Singlet Oxygen Detection

First, 5 μL of the QD@Phage solution (10^6 PFU/mL in SM buffer) was added to 500 μL of DPBF solution (30 μM, dissolved in DMF) in cuvette, and DPBF+PBS solution was served as a control. The mixture was irradiated under visible light (5W) in 16 min while recording the UV-vis spectra using UV-vis spectrometer every 4 mins. In paralell, they were also measured under dark condition.

Adsorption rate

The adsorption curve assay was performed to determine the phage adsorption rate. Briefly, GFP-*P. aeruginosa* was grown in 10 mL Luria-Bertani (LB) broth at 37 °C under sacking conditions (160 rpm/min) to reach an OD600 of 0.4 (corresponding to approx. 10^8 CFU/mL). Then QD@Phage (10^6

PFU/mL) was mixed with the bacterial suspension at MOI = 0.1 and incubated at 37°C. Aliquots of 100 μL were taken at 5 min intervals during 25 min and diluted in 4.4 mL LB broth containing 0.5 mL of chloroform. The number of non-adsorbed phage particles at each incubation time was determined by the double agar overlay method.

Intracellular ROS assay

The 2′,7′-dichlorodihydrofluorescein diacetate (DCF-DA) Assay Kit was performed to detect the intracellular ROS level of the bacteria induced by QD@Phage. Briefly, GFP-*P. aeruginosa* cells were treated with phage, QD, and QD@Phage under dark and light conditions for 90 min at 37 °C. After washing with PBS, the treated samples were co-incubated with 10 μM DCF-DA probe for 30 min in the dark, followed by detecting fluorescence intensity using plate reader (Ex/Em = 492/517 nm).

Specific antibacterial test

GFP-*P. aeruginosa* and MRSA bacterial mixture suspensions (~10^6 CFU/mL) in LB were used for the specific antibacterial tests. The 100 μL of mixture bacterial suspension was firstly mixed with 900 μL of ~10^5 PFU/mL QD@Phage in centrifuge tubes and incubated for 30 min under dark at 37 ^{o}C, and then they were incubated under light for another 60 min. 100 μL of mixture bacterial suspensions were then serially diluted and added onto the LB agar plates. After incubation at 37 °C for 24 h, the mixture bacterial colonies were distinguished and recorded by Gel Documentation System Bio-Rad (Chemidoc MP).

Binding and imaging ability

GFP-*P. aeruginosa* suspensions (10^8 CFU/mL) were treated with QD and QD@Phage (almost equivalent QD contents (determined by fluorescence intensity using plate reader)) for 30 mins at 37 ℃ under dark condition. Samples were then washed three times to remove the unbound QD using Ultrafree-MC Centrifugal Filter (Merck) via centrifugation. Then, the mixture was resuspended in PBS for obtaining fluorescent images on confocal laser scanning microscope (CLSM, TCS SP8, Leica, Germany).

Antibacterial test

Typically, 10 μL of QD@Phage solution (10^6 PFU/mL) was introduced into 90 μL of ~10^5 CFU/mL bacterial suspensions. After incubation under dark at 37 ℃ for 30 minutes, the bacterial suspensions were incubated at 37 ℃ for another 60 min under darkness conditions or with visible light irradiation

by a 5 W LED lamp with a power density of 0.1 W/cm^2. Experimental groups including phage (10^6 PFU/mL), QD, QD@Phage and gentamicin (8 μg/mL, minimum bactericidal concentration) with different conditions carried out as comparison. At last, the bactericidal ratio for all groups were evaluated via agar plate counting. The bacterial suspensions were diluted and cultured on agar plates for quantification of bacterial counts after overnight incubation at 37 °C. The bactericidal ratio of the examined samples was calculated by the following equation: R=(100-Is/Ic) ×100%, where R represents the bactericidal ratio, Ic represents average amount of control group, and IS represents the average amount of bacteria treated with different groups. The antibacterial activity of QD@Phage was evaluated against planktonic of GFP-*P. aeruginosa* by the time-killing assay. Briefly, the planktonic cells (10^5 CFU/mL) were incubated with QD@Phage at 37 ℃ for 0, 30, 60 and 90 min. Subsequently, ten-fold serial dilutions of cells were plated onto LB agar and incubated for 18-24 h at 37°C for cell counting.

Biofilm eradication test.

GFP-*P. aeruginosa* biofilm was cultured by adding 200 μL GFP-*P. aeruginosa* suspension (10^5 CFU/mL in LB medium) was placed in 8-well slides, then they were cultured at 37 °C. Twenty-four hours later, the medium was removed, thereby, the unattached bacteria was gently washed away with sterile PBS three times, and the mature biofilm on 8-well slides was harvested. Next, the prepared biofilm was treated with QD@Phage for 30 min under dark at 37 ^{o}C, and then it was irradiated by visible light overnight at 37 ^{o}C. Finally, the sessile cells were diluted and cultured on agar plates for overnight to count the bacterial colonies. The anti-biofilm activity of QD@Phage was evaluated against GFP-*P. aeruginosa* biofilm by the time-killing assay. 24 h-old biofilm was incubated with QD@Phage at 37 ℃ for 0, 6, 12 and 24 h, subsequently, ten-fold serial dilutions of the biofilm-embedded cells were plated onto LB agar and after 18-24 h incubation at 37°C for cell-counting.

To calculate the antibiofilm efficiency, the following formula was used: C =(100–B/A) ×100%, where C indicates antibiofilm efficiency, A is the number of colony forming units (CFUs) in the control group (PBS), and B is the number of CFUs in experimental groups. Furthermore, crystal violet staining assay was used to investigate the biofilm biomass. Briefly, the treated biofilms were stained with crystal violet (0.1%, 10 min), then they were washed with PBS three times. Thereafter, the crystal violet was dissolved into 200 μL of ethanol. The biomass of the biofilm was evaluated by measuring

the absorbance at 595 nm. Experimental groups including phage (10^6 PFU/mL), QD, and QD@Phage with different conditions were carried out as comparison.

In addition, 200 μL of QD@Phage (10^6 PFU/mL) was introduced into the mature biofilm in 8-well slides for 3D confocal observation. After incubation at 37 ℃ for 30 min, the visible light irradiation was conducted for overnight. Subsequently, the biofilms were gently washed by PBS three times and co-stained with propidium iodide (PI) according to the protocol (DEAD BacLight Bacterial Viability Kit, L7012, Molecular Probes). After sample fixation with 4% paraformaldehyde (fresh prepared in PBS, pH 7.2) at room temperature for 20 min, the 3D Z-stack fluorescent and orthogonal-stack images were acquired on the SP8 lighting confocal laser scanning microscope (Leica, Germany). The fluorescent intensities were analyzed by ImageJ pro.

Cytotoxicity

Human A549 and HaCat cell lines were chosen as models to investigate the cells toxicity. 1×10^5 cells were grown in Eagle's minimal essential medium (DMEM) supplemented with 10% FCS and 1% Pen/Strep (100 U/mL penicillin; 100 μg/mL streptomycin) in a 96-well plate at 37 °C and 5% CO_2. After that, 10 μL of QD@Phage and PBS were mixed with the resuspended cells (100 μL) to investigate cell toxicity. For the QD@Phage-treated cells, they were exposed to visible light irradiation for overnight. For the control group (PBS), cells were kept in darkness for overnight. Cell viability was then analyzed by CCK-8 method. Detailly, 100 μL of different treated cells containing 10 μL CCK-8 solution were added into each well and incubated at 37 °C for 2 h, the absorbance at 450 nm was recorded using a Plate Reader Tecan (Multiscan GO, Thermo Scientific).

***In-vivo* antibacterial activity evaluation**

All animal experiments were performed humanely in compliance with guidelines reviewed by the animal ethics committee of Shanghai Sixth People's Hospital (approval number: 2021-0288). For *in-vivo* experiments, healthy adult BALB/c mice (500 g, male, Chengdu Dossy Biological Technology Co. Ltd. (China)) were used, and they were randomly divided into four groups: (1) control group (PBS); (2) phage group; (3) QD light group; (4) QD@Phage light group (n= 3 per group). After anesthetized with 2% sodium pentobarbital, a small round incision (1cm in diameter) on the dorsal epidermis of mice was firstly infected by 100 μL of GFP- *P. aeruginosa* (1×10^8 CFU/mL) for 24 h to establish *in-vivo* biofilm. After cultured for 1 day, QD@Phage (200 μL, 10^6

PFU/mL, only one dosage over the whole treatment) was dropped onto the infected wound. Then, the wound was irradiated by a xenon lamp (60 W, 1 m high, 2 h) with the power density of 0.1 W/cm^2 to achieve antibacterial disinfection. During the antibacterial process, fluorescence images were measured by a VISQUE In Vivo Smart-LF (Vieworks, Anyang, South Korea) under inhalation anesthesia every two days with an excitation of 488 nm and an emission of 509 nm. For comparison with activated QD@Phage, groups of phage, pristine QD light and PBS were also conducted to evaluate the *in-vivo* biofilm eradication efficacy. The mice in these different groups were photographed and the size of infected wounds were dynamically monitored through measurements to evaluate the healing efficacy. The wound was photographed with reference ruler and the size was processed by Image J. After treatment for 9 days, all mice were sacrificed, and the tissues of infected wound area were harvested for pathological histology analysis.

Hematoxylin–Eosin and Masson staining

The extracted tissues were fixed in 4% paraformaldehyde, paraffin embedded, and sectioned for H&E and Masson staining using standard protocols. The conventional paraffin sections were soaked in xylene for 30 min, dehydrated with 100, 95, 85, and 75% ethanol solutions for 5 min, followed by water washing, hematoxylin staining for 10 min, soaking in 1% hydrochloric acid, and rinsing with distilled water. Eosin staining (Dakwei, Beijing, China) for 3 min, 75, 85, 95, and 100% ethanol solution for 2 min, xylene transparent treatment, drop neutral resin cover glass, and placed under an inverted microscope (Leica, DM300) to observe the results. Masson staining was used to observe collagen deposition in the wound tissue. Paraffin sections of the wound tissue were dewaxed and hydrated. Ponceau acid fuchsin solution was used for staining for 5–10 min. After washing with distilled water, 1% phosphomolybdic acid solution was used for staining for 5 min. After removing the dye solution, the sections were stained with aniline blue for 5 min, washed with distilled water, and treated with 1% glacial acetic acid for 1 min. The samples were dehydrated with 95% ethanol and anhydrous ethanol, transparent with xylene, and sealed.

Statistical Analysis

All experimental data were expressed as the mean ± standard deviation. Statistical analyses were conducted using GraphPad Prism 9 software (GraphPad Software, Inc., San Diego, CA, USA). The mean values of two groups were compared using t test and one-way ANOVA followed by Dunnett's post-hoc test. A probability (P) value of ≤ 0.05 was considered statistically significant.

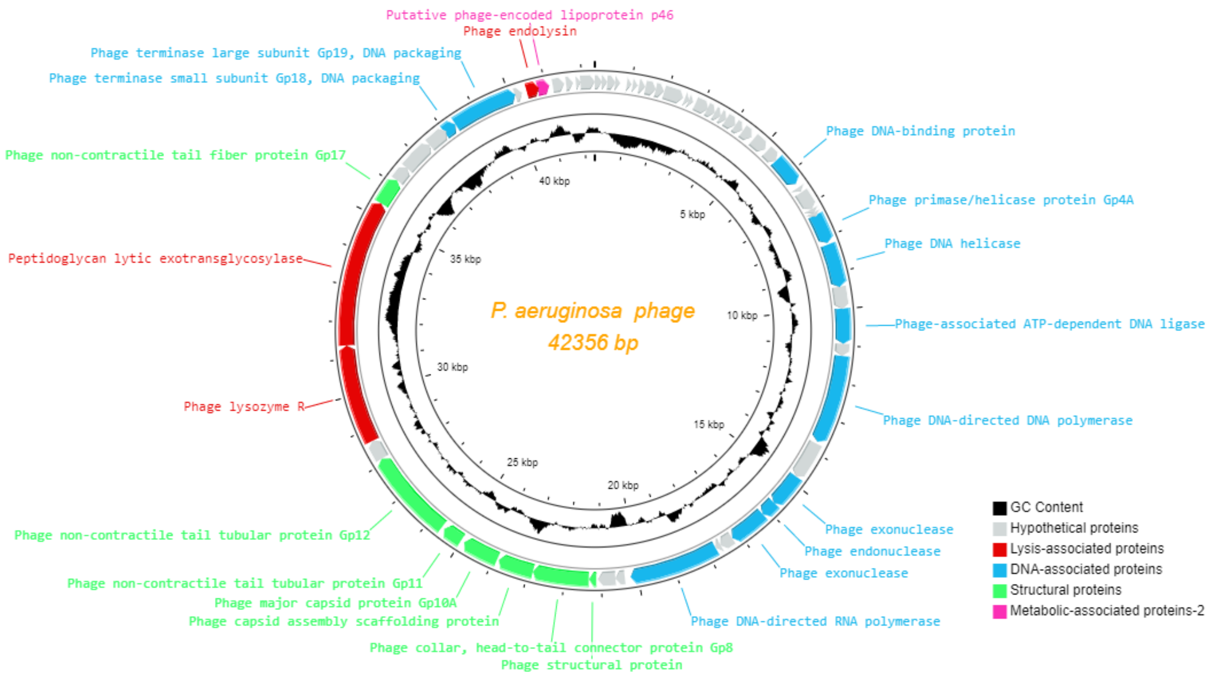

Figure S1. Circular representation of the genome of the *P. aeruginosa* phage. The outer circle indicates the gene coding region. The colour of each gene coding region refers to the functional category: hypothetical proteins (grey), lysis-associated proteins (red), DNA-associated proteins (blue), structural proteins (green) and metabolic-associated proteins (pink). The black inner circle indicates the GC content, and the most inner ring shows genome location (kbp). Genome size: 42,356 bp with a GC content of 62.3%. BLASTn identified most similarity to *P. aeruginosa* phage phiNFS (Genbank accession number NC_047852; 99% query cover; 96.84% sequence identity), therefore being classified as a new phage member within the *Phikmvvirus* genus. The undesirable genes did not detect from the described database.

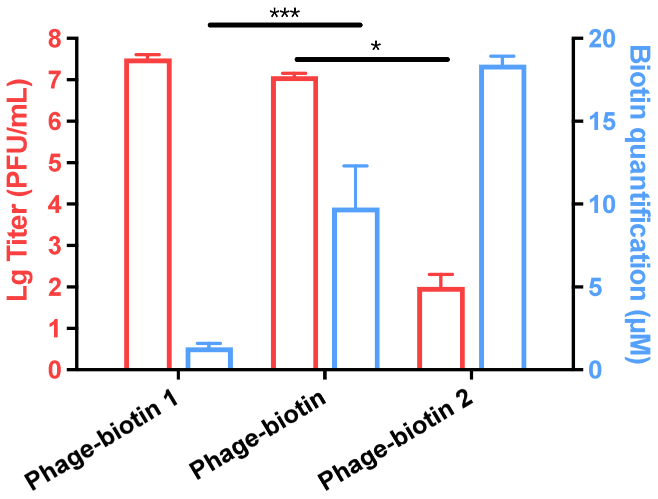

Figure S2. Biotin quantifications and corresponding titers for different series of Phage-biotin. Phage-biotin 1, phage-biotin and phage-biotin 2 synthesized with different feeding doses of 87.87 μM, 878.7 μM and 1757.6 μM of Biotin-NHS, respectively. The data are presented as mean ± standard deviation (SD), n=3. *P < 0.05, ***P < 0.001 by one-way ANOVA followed by Dunnett's post-hoc test.

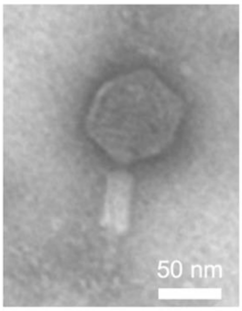

Figure S3. A representative transmission electron microscope (TEM) image of Phage. Scale bar: 50 nm.

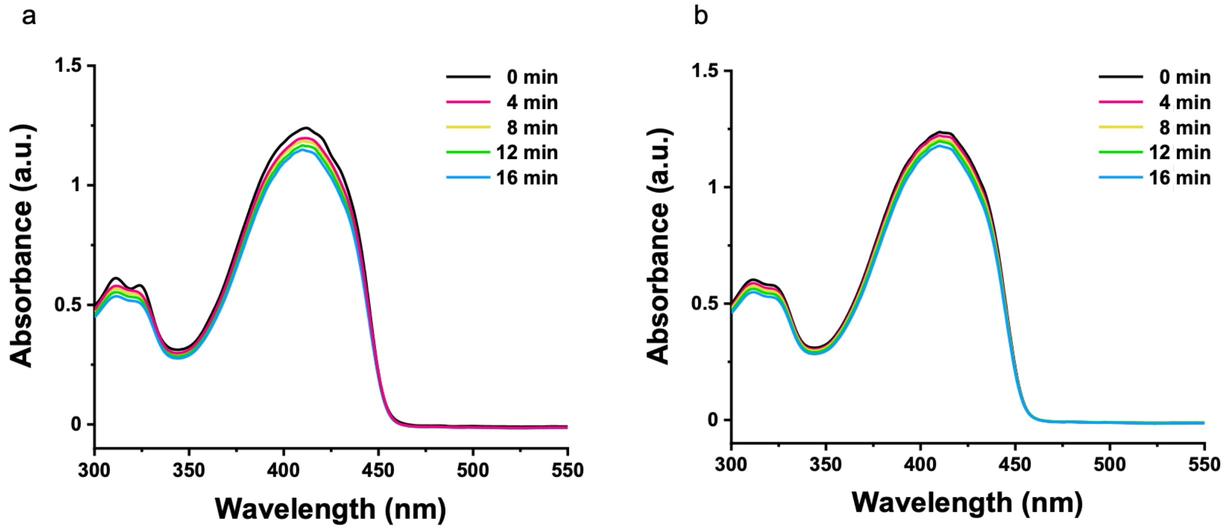

Figure S4. UV–vis absorption spectra of (a) DPBF and (b) DPBF + QD@Phage under dark incubation, respectively.

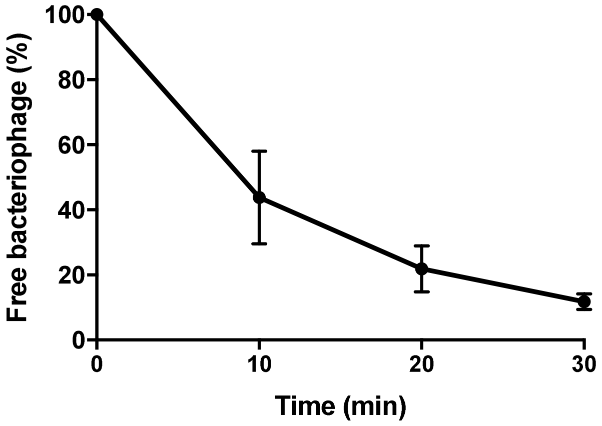

Figure S5. Adsorption curve of QD@Phage assessed on GFP- *P. aeruginosa*. Data are expressed as the mean ± standard deviation (SD), n=3. The free QD@Phage decrease 88.7% from the supernatant with prolongation of the incubation time from 0 to 30 min when used the characteristic of phage adsorption rate.

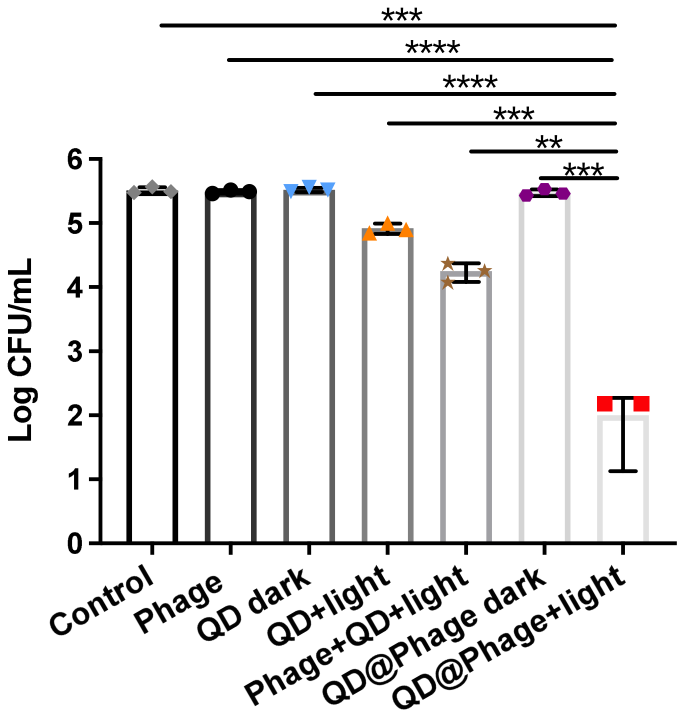

Figure S6. The cell count of phage, QD dark, QD+light, Phage+QD+light, QD@Phage dark and QD@Phage+light against planktonic of GFP-*P. aeruginosa*, respectively. The PBS treatment served as the control group. The data are presented as mean ± SD, n=3. ***P* < 0.01, ****P* < 0.001, *****P* < 0.0001 by one-way ANOVA followed by Dunnett's post-hoc test. In QD@Phage+light group, one group showed no colony after treatment, therefore, the data is not present.

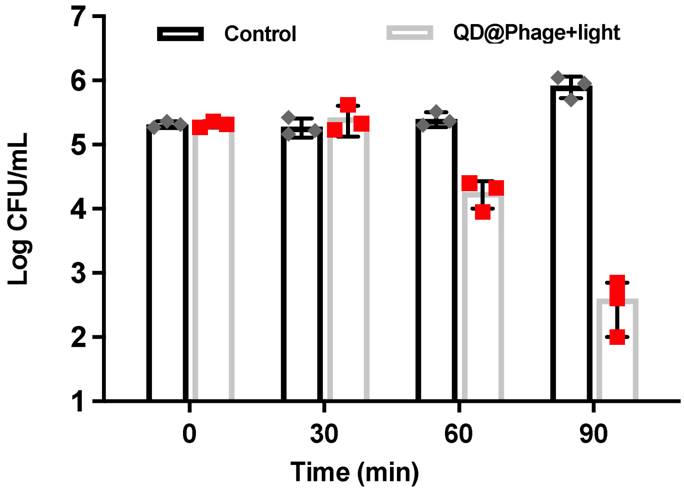

Figure S7. Bacterial cell count of planktonic GFP-*P. aeruginosa* treated with QD@Phage (10^6 PFU/mL) and untreated monitored at 30 min intervals during 90 min by the time-killing assay. The PBS treatment served as the control group. Data are expressed as mean ± SD, n=3.

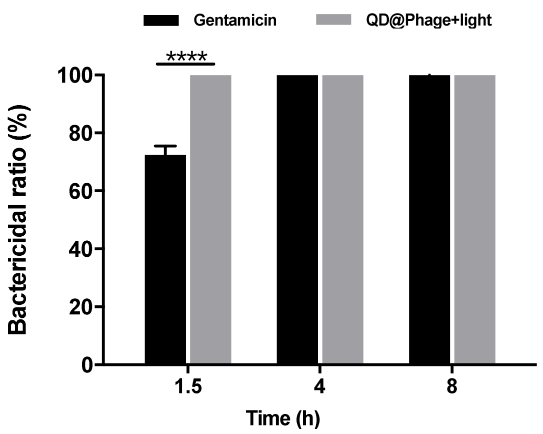

Figure S8. Bactericidal ratio of gentamicin and QD@Phage with visible light irradiation at different incubation time. The data are presented as mean ± SD, n=3. ****P < 0.0001 by t test.

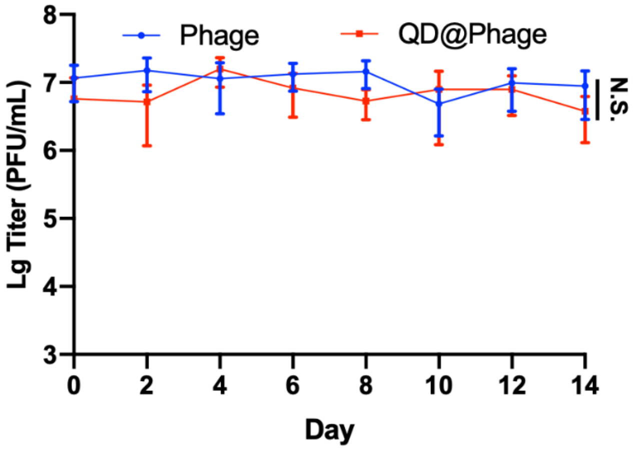

Figure S9. The infectivity stability of phage and QD@Phage. The data are presented as mean ± SD, n=3. N.S. represents no significance, P > 0.05 by t test.

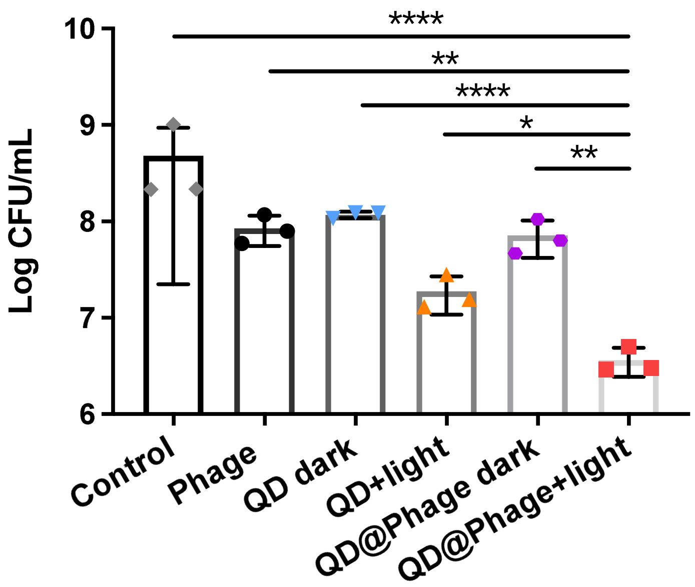

Figure S10. The cell count of phage, QD dark, QD+light, QD@Phage dark and QD@Phage+light against biofilm of GFP-*P. aeruginosa*, respectively. The PBS treatment served as the control group. The data are presented as mean ± SD, n=3. **P* < 0.05, ***P* < 0.01, *****P* < 0.0001 by one-way ANOVA followed by Dunnett's post-hoc test.

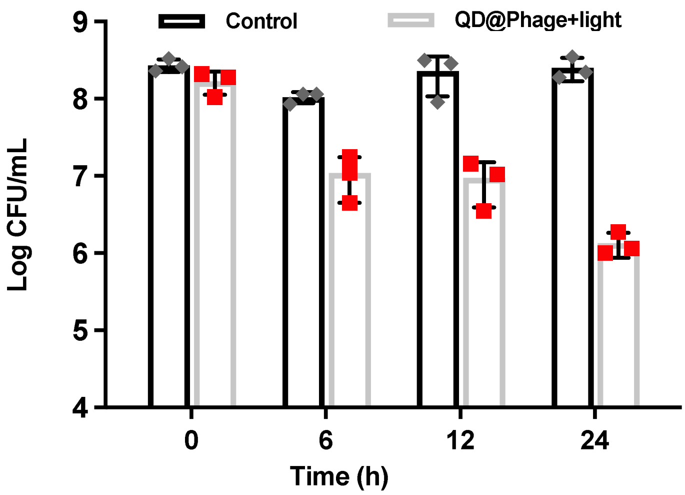

Figure S11. Bacterial cell count of GFP-*P. aeruginosa* biofilms treated with QD@Phage (10^6 PFU/mL) and untreated monitored at different time during 24 h. The PBS treatment served as the control group. Data are expressed as mean ± SD, n=3.

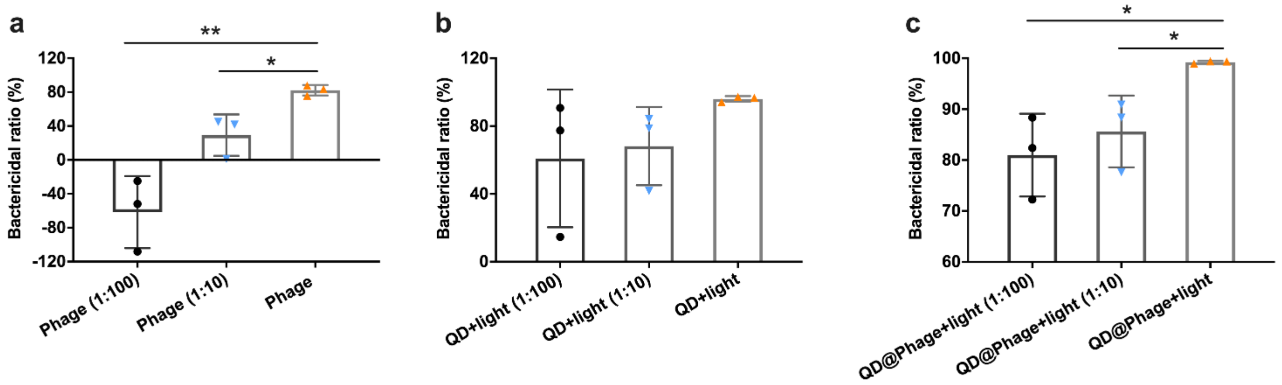

Figure S12. The biofilm bactericidal ratio of different concentrations of (a) phage, (b) QD light, and (c) QD@Phage light against GFP-*P. aeruginosa*, respectively. 1:10 and 1:100 refered to 10 and 100 times dilution of samples, respectively. The data are presented as mean ± SD, n=3. **P* < 0.05, ***P* < 0.01 by one-way ANOVA followed by Dunnett's post-hoc test for data in (a) and (c).

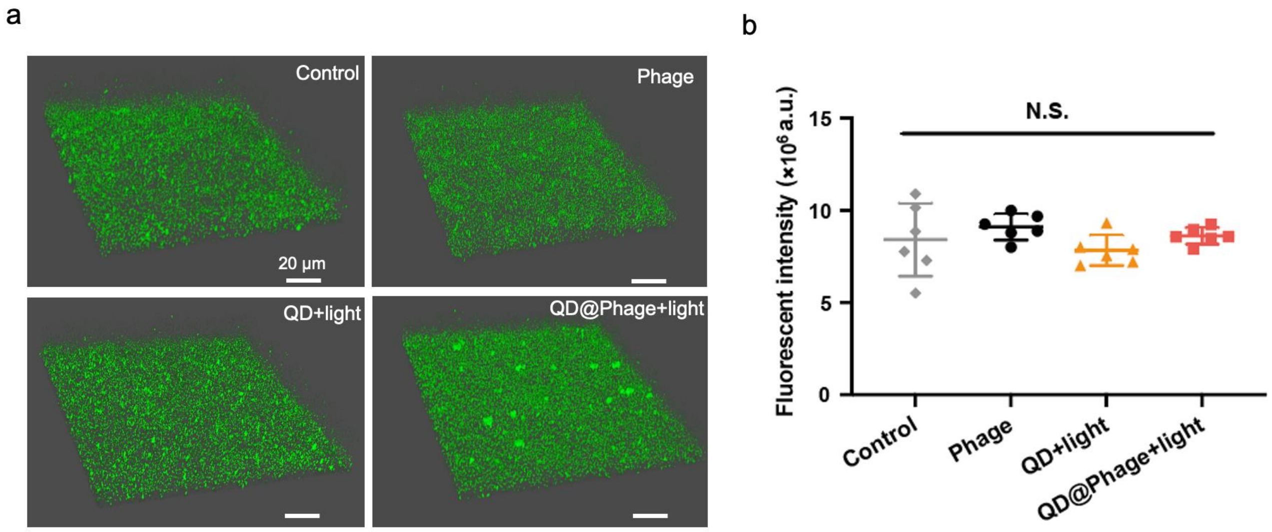

Figure S13. (a) The confocal microscopy images of GFP-*P. aeruginosa* in the biofilm structure after treatments of PBS, phage, QD and QD@Phage under different conditions excited by 488 nm and (b) corresponding semiquantitative statistics of fluorescent intensity of alive cells in the biofilm structure. The data are presented as mean ± SD, n=6. N.S. represents no significance, *P* > 0.05 by one-way ANOVA followed by Dunnett's post-hoc test for data in (b). Scale bar: 20 μm. The PBS treatment served as the control group. We noticed no significant difference among groups, which might be attributed to the GFPs still presented in *P. aeruginosa* cell after treatment.

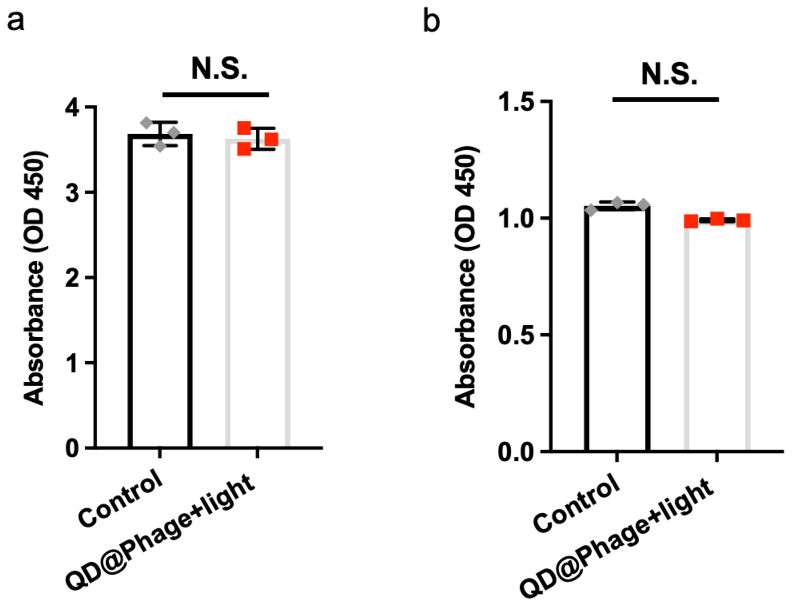

Figure S14. Cell Counting kit-8 (CCK-8) analysis of (a) human A549 cell and (b) human keratinocyte cell. The PBS treatment served as the control group. The data are presented as mean ± SD, n=3. N.S. represents no significance, $P > 0.05$ by t test.

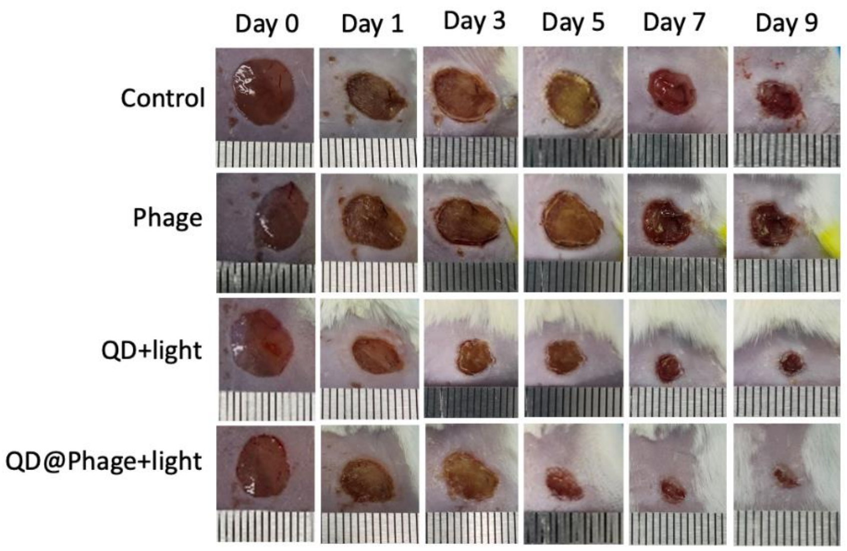

Figure S15. Typical wound photos during healing of groups treated with phage, QD and QD@Phage. The PBS treatment served as the control group.

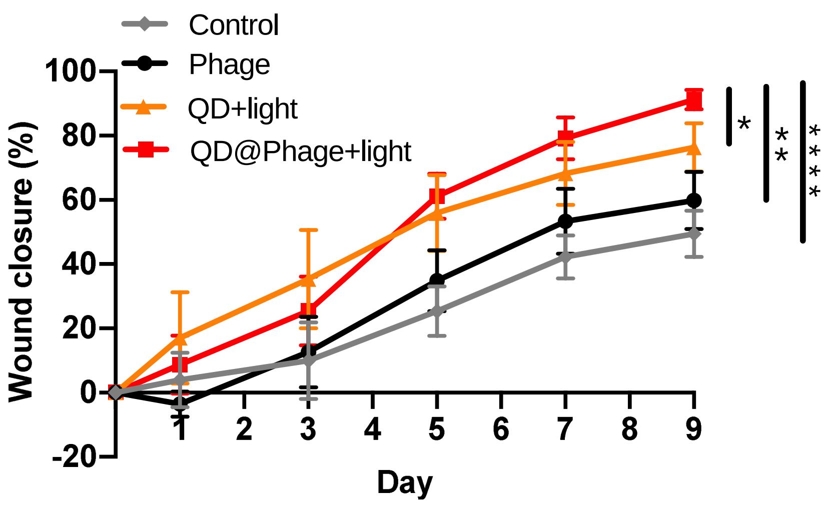

Figure S16. The wound-closure rate after the treatments of phage, QD and QD@Phage under different conditions. The data were recorded via a digital camera and analyzed by ImageJ. The PBS treatment served as the control group. The data are presented as mean ± SD, n=3. *P < 0.05, **P < 0.01, ****P < 0.0001 by one-way ANOVA followed by Dunnett's post-hoc test.

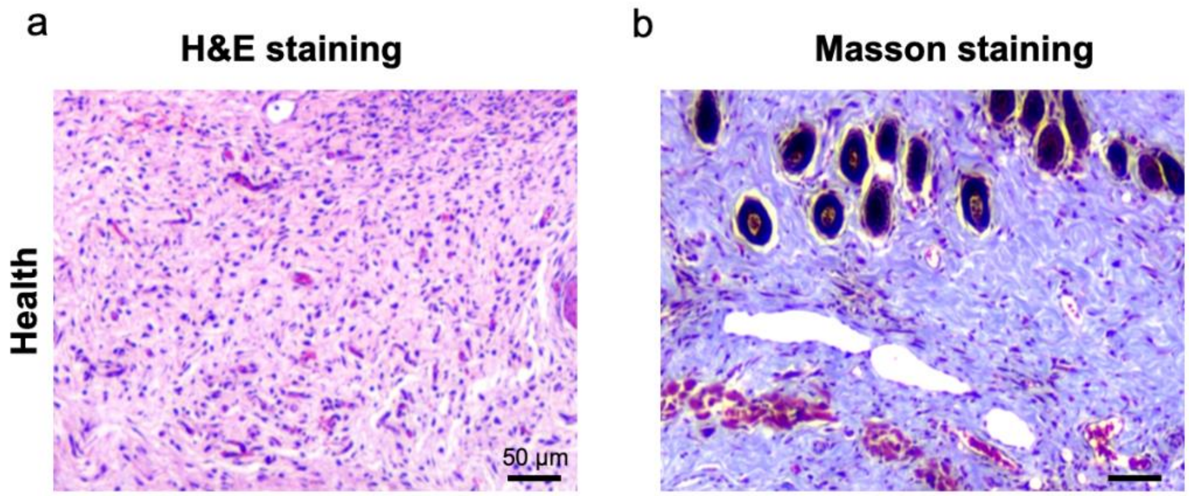

Figure S17. Histological photomicrographs of the healthy epidermal histological sections after (a) H&E and (b) Masson staining. Scale bar: 50 μm.
